# Diagnóstico Pré-Natal de Duplo Arco Aórtico

**DOI:** 10.36660/abc.20190310

**Published:** 2021-02-02

**Authors:** Natália Noronha, Angie Hobbs, Patricia Caldas

**Affiliations:** 1 Bristol Royal Hospital for Children Bristol Reino Unido da Grã-Bretanha Bristol Royal Hospital for Children, Bristol - Reino Unido da Grã-Bretanha

**Keywords:** Cardiopatias Congênitas/diagnóstico, Cardiopatias Congênitas/cirurgia, Aorta Torácica/diagnosis, Heart Defects Congenital/surgery, Aberrações Cromossômicas, Ultrassonografia/métodos, Ecocardiografia/métodos, Paralisia das Cordas Vocais, Broncomalácia/congênito

## Introdução

As anomalias congênitas do arco aórtico afetam 1-2% da população e compreendem uma grande variedade de anormalidades na posição e / ou padrão de ramificação do arco aórtico.^1^ O duplo arco aórtico (DAA) representa 1-2% de todas as anomalias do arco aórtico e é caracterizado pela persistência dos arcos aórticos embrionários esquerdo e direito.^2^ A anomalia pode ser encontrada isoladamente ou, com menor frequência, em associação com outras anormalidades cardiovasculares ou cromossômicas. O diagnóstico pré-natal de DAA pode ser desafiador, pois sua diferenciação de outras anormalidades do arco, como arco aórtico direito com canal arterial esquerdo ou com artéria subclávia esquerda aberrante, nem sempre é clara. Os autores descrevem um caso de diagnóstico pré-natal de DAA.

## Relato de Caso

Uma primigesta de 26 anos foi encaminhada para uma revisão de cardiologia fetal com 23 + 3 semanas de gestação devido a uma imagem anormal dos três vasos e traqueia (3VT) em um exame de ultrassonografia morfológica de rotina com 20 semanas de gravidez. Ela não estava tomando medicamentos e não havia histórico pessoal ou familiar relevante.

**Figura 1:**
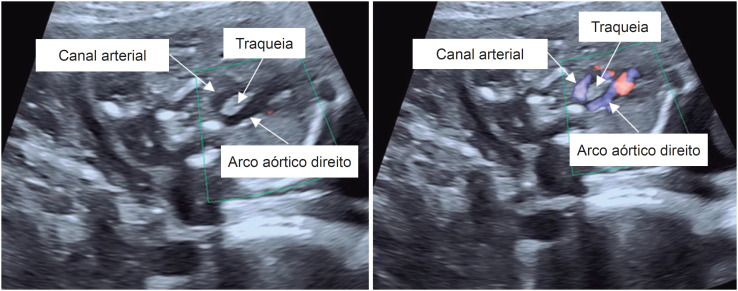
Ecocardiograma fetal (projeção dos três vasos e traqueia) mostrando um canal arterial esquerdo e direito circundando completamente a traqueia sem (painel esquerdo) e com (painel direito) mapeamento de fluxo em cores.

**Figura 2:**
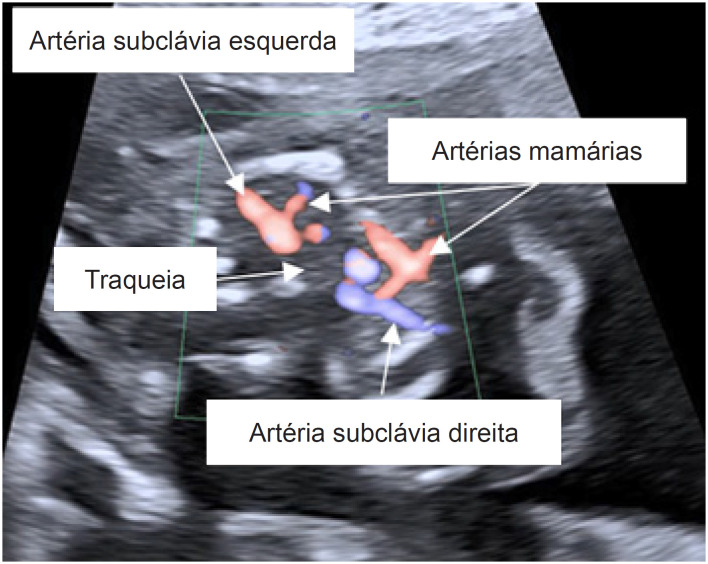
Ecocardiograma fetal (imagem axial de fluxo em cores) mostrando as artérias subclávias direita e esquerda originando-se dos arcos aórticos esquerdo e direito, respectivamente.

O ecocardiograma fetal mostrou projeção de quatro câmaras e vias de saída normais. Na projeção de 3VT, entretanto, o canal arterial foi visto do lado esquerdo da traqueia e o arco aórtico do lado direito, confirmando a presença de um arco aórtico direito com canal arterial esquerdo (Figura 1). Com uma imagem mais próxima, notou-se uma estrutura menor do lado esquerdo da traqueia, circundando a mesma completamente. Para confirmação do diagnóstico de DAA, as artérias subclávias foram localizadas e cada uma foi vista originando-se do respectivo arco aórtico (Figura 2). Não havia sinais de obstrução em nenhum dos arcos aórticos. Nenhuma outra anormalidade cardíaca ou extracardíaca foi encontrada. Dada a possível associação de DAA com anormalidades cromossômicas, particularmente a microdeleção 22q11.2, o casal foi aconselhado a se submeter a testes invasivos, os quais eles recusaram.

**Figura 3:**
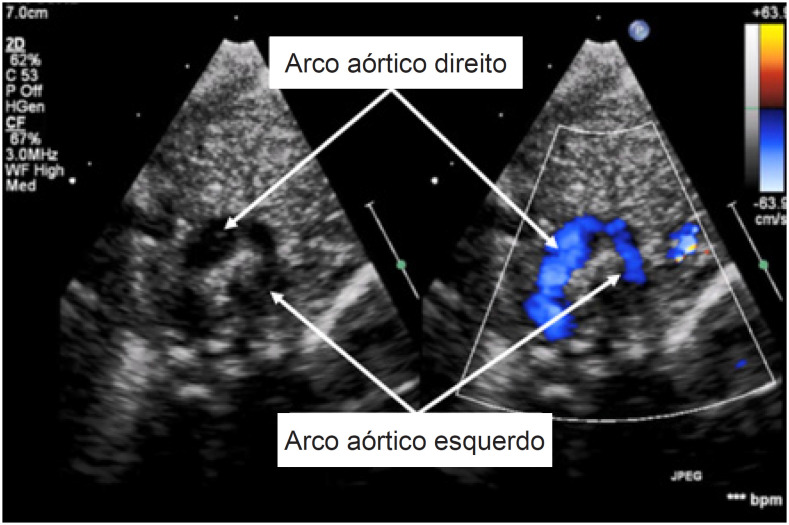
Ecocardiograma transtorácico - projeção paraesternal alta (2D e mapeamento de fluxo em cores) mostrando o arco aórtico direito dominante e o menor esquerdo.

**Figura 4:**
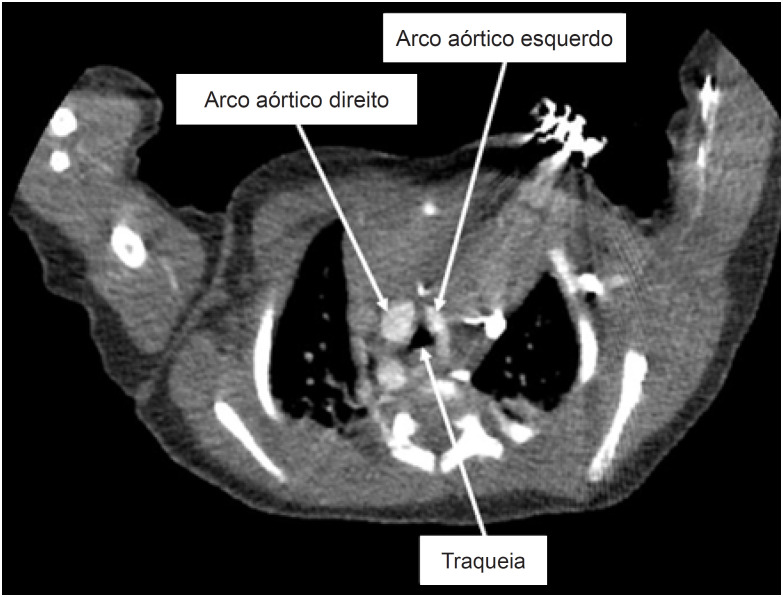
Tomografia computadorizada - imagem axial mostrando o arco aórtico direito dominante e o menor esquerdo.

Após o parto, um exame de imagem pós-natal confirmou os achados pré-natais (Figura 3). Aos 2 meses de idade, os pais notaram estridor leve intermitente. O paciente foi encaminhado para uma tomografia computadorizada cardíaca, que confirmou o diagnóstico de duplo arco aórtico com atresia do arco distal esquerdo (Figura 4). O paciente foi submetido à separação cirúrgica do arco esquerdo aos 3 meses de idade, complicada por paralisia de prega vocal esquerda.

O paciente tem atualmente 5 meses de idade, apresenta estridor intermitente por broncomalácia e paralisia de prega vocal esquerda e é alimentado por sonda nasogástrica.

## Discussão

O DAA é o substrato mais frequente para um anel vascular e pode resultar em sintomas respiratórios e / ou digestivos desde tenra idade. Na maioria dos casos, um dos arcos é dominante, mais frequentemente o direito (em pelo menos 75% dos casos).^2^ Pode haver um segmento de atresia em um ou vários locais em qualquer um dos dois arcos, geralmente o esquerdo,^1^^,^^3^ como aconteceu em nosso caso. O DAA resulta em sintomas respiratórios como estridor, episódios de asfixia e infecções recorrentes do trato respiratório em 91% dos pacientes. Os sintomas gastrointestinais, por outro lado, ocorrem em 40% dos casos e incluem vômitos, intolerância alimentar em lactentes e disfagia em crianças maiores e adultos.^2^^,^^4^

O diagnóstico de DAA pode ser feito através da projeção de 3VT descrita por Yagel et al.,^5^ Nesta projeção, o arco aórtico normal (esquerdo) é observado à esquerda da linha média e da traqueia. O canal arterial é visto lateralmente no seu lado esquerdo. O arco aórtico e o canal arterial convergem então em uma estrutura em forma de V que continua como a aorta descendente. O terceiro vaso que compreende a projeção de 3VT é a veia cava superior, que é vista à direita da linha média. Em um arco aórtico esquerdo normal, nenhuma estrutura vascular importante é vista cruzando a traqueia posteriormente. Por outro lado, no DAA, a projeção de 3VT representa um arco aórtico esquerdo e direito, formando um anel vascular que circunda completamente a traqueia. Este anel, junto com o canal arterial, forma a figura de um “6” ou um “9” (também descrito como formato de tridente) em vez da estrutura clássica em forma de V descrita acima. A presença de fluxo anterógrado em ambos os arcos e no canal arterial pode ser confirmada pelo mapeamento de fluxo em cores. Este último também deve ser utilizado para confirmar ou excluir obstrução ao fluxo em qualquer um dos arcos.

Pode ser difícil diferenciar o DAA de outras anormalidades do arco aórtico, como arco aórtico direito com canal arterial esquerdo na projeção de 3VT. Nessa situação, a identificação da origem das artérias subclávias pode auxiliar no diagnóstico diferencial. Se cada uma das artérias subclávias for vista surgindo dos arcos aórticos esquerdo e direito (para os lados esquerdo e direito da traqueia, respectivamente), o diagnóstico de DAA pode ser estabelecido.

Foi relatado que o reparo cirúrgico precoce do DAA elimina os sintomas em mais de 70% dos casos, embora a limitação do fluxo de ar possa persistir devido à estenose traqueal residual.^2^ Em uma revisão de 183 pacientes com anéis vasculares submetidos à correção cirúrgica,^6^ 2 pacientes necessitaram de traqueostomia por grave compressão distal da traqueia e um paciente apresentou paralisia de prega vocal esquerda verdadeira, como ocorreu em nosso caso.

Embora desafiador, o diagnóstico pré-natal de DAA permite uma caracterização oportuna do anel vascular e facilita o planejamento da intervenção cirúrgica antes ou logo após o desenvolvimento dos sintomas. Embora os sintomas possam não ser resolvidos imediatamente, uma separação precoce do DAA é crucial para prevenir sequelas de longo prazo de compressão traqueobrônquica e dificuldades de alimentação.^2^
