# Supplementary material for: Diagnóstico Pré-Natal de Duplo Arco Aórtico
Source: Arq Bras Cardiol. 2021 Feb 2;116(2 Suppl 1):4–7. [Article in Portuguese] doi: 10.36660/abc.20190310 (PMC8118628; doi:10.36660/abc.20190310)
Supplement: Translation

# Translation: Antenatal Diagnosis of Double Aortic Arch

**Authors:** Natália Noronha, Angie Hobbs, Patricia Caldas

**Affiliations:** 1 Bristol United Kingdom Bristol Royal Hospital for Children, Bristol - United Kingdom

**Keywords:** Heart Defects Congenital/diagnosis, Heart Defects Congenital/surgery, Aorta Thoracic, Chromosome Aberrations, Ultrasonography/methods, Echocardiography/methods, Vocal Cord, Paralysis, Bronchomalacia/congenital

## Introduction

Congenital anomalies of the aortic arch affect 1-2% of the population and comprise a wide variety of abnormalities in the position and/or branching pattern of the aortic arch.^1^ Double aortic arch (DAA) represents 1-2% of all aortic arch anomalies and is characterized by persistence of both left and right embryonic aortic arches.^2^ It can be found either in isolation or, less frequently, in association with other cardiovascular or chromosomal abnormalities. Antenatal diagnosis of DAA may be challenging, as its distinction from other arch abnormalities like right aortic arch with left arterial duct or with an aberrant left subclavian artery is not always straightforward. The authors describe a case of antenatal diagnosis of DAA.

## Case Report

A 26-year-old primigravida was referred for a fetal cardiology review at 23+3 weeks of gestation due to an abnormal three vessels and trachea (3VT) view in her routine 20-week anomaly scan. She was on no medications and there was no relevant personal or family history.

The fetal echocardiogram showed normal four-chamber view and outflow tracts. In the 3VT view, however, the arterial duct was seen on the left side of the trachea and the aortic arch was on the right side, confirming the presence of a right aortic arch with a left sided arterial duct (Figure 1). On a closer look, a smaller structure was seen on the left side of the trachea, completely encircling the latter. To confirm the diagnosis of DAA, the subclavian arteries were located and each one was seen to arise from the respective aortic arch (Figure 2). There were no signs of obstruction in either aortic arch. No other cardiac or extracardiac abnormalities were found. Given the possible association of DAA with chromosomal abnormalities, particularly the 22q11.2 microdeletion, the couple was advised to undergo invasive testing, which they declined.

**Figure 1:** Fetal echocardiogram (three vessels and trachea view) showing a left arterial duct and a right aortic arch completely encircling the trachea without (left panel) and with (right panel) color flow mapping.

**Figure 2:** Fetal echocardiogram (color flow axial image) showing the left and the right subclavian arteries arising from the left and the right aortic arches, respectively.

After the delivery, a postnatal scan confirmed the antenatal findings (Figure 3). At 2 months of age, his parents noticed mild intermittent stridor. The patient was referred for a cardiac computerized tomography, which confirmed the diagnosis of a double aortic arch with atresia of the distal left arch (Figure 4). The patient underwent surgical division of the left arch at 3 months of age, which was complicated by left vocal cord palsy.

**Figure 3:** ETransthoracic echocardiogram - high parasternal view (2D and color flow mapping) showing the dominant right and the smaller left aortic arch.

**Figure 4:** Computerized tomography scan - axial image showing the dominant right and the smaller left aortic arch.

The patient is currently 5-months-old, has intermittent stridor due to bronchomalacia and left vocal cord palsy, and is fed by a nasogastric tube.

## Discussion

DAA is the most frequent substrate for a vascular ring and might result in respiratory and/or digestive symptoms from an early age. In the majority of cases, one of the arches is dominant, more frequently the right arch (at least 75% of cases).^2^ There may be an atretic segment in one or several locations in either arch, usually the left,^1^^,^^3^ as happened in our case. DAA results in respiratory symptoms like stridor, choking episodes and recurrent respiratory tract infections in 91% of patients. Gastrointestinal symptoms, on the other hand, occur in 40% of cases and include vomiting, feeding intolerance in infants and dysphagia in older children and adults.^2^^,^^4^

The diagnosis of DAA can be made in the 3VT view described by Yagel et al.^5^ In this view, the normal (left) aortic arch is observed on the left of the midline and the trachea. The arterial duct is seen laterally on its left side. The aortic arch and arterial duct then converge into a V-shaped structure that continues as the descending aorta. The third vessel that comprises the 3VT view is the *superior vena cava*, which is seen on the right of the midline. In a normal left aortic arch, no major vascular structures are seen to cross the trachea posteriorly. Conversely, in DAA the 3VT view depicts both a left and a right aortic arch, forming a vascular ring that completely encircles the trachea. This ring, together with the arterial duct, forms the figure of either a “6” or a “9” (also described as a trident shape) instead of the classic V-shaped structure described above. The presence of antegrade flow in both arches and in the arterial duct can be confirmed by color flow mapping. The latter should also be used to confirm or exclude obstruction to flow in any of the arches.

It may be difficult to distinguish DAA from other aortic arch abnormalities, such as right aortic arch with left arterial duct in the 3VT view. In this situation, the identification of the origin of the subclavian arteries may aid in the differential diagnosis. If each of the subclavian arteries is seen to arise from the left and right aortic arches (to the left and to the right side of the trachea, respectively), the diagnosis of DAA can be established.

Early surgical repair of DAA has been reported to eliminate symptoms in over 70% of cases, although airflow limitation might persist due to residual tracheal stenosis.^2^ In a review of 183 patients with vascular rings who underwent surgical repair,^6^ 2 patients required tracheostomy due to severe distal tracheal compression and one patient had true left vocal cord palsy, as happened with our case.

Albeit challenging, antenatal diagnosis of DAA enables a timely characterization of the vascular ring and facilitates planning of surgical intervention before or shortly after symptoms develop. Although symptoms may not be relieved immediately, an early division of the DAA is crucial to prevent long-term sequelae of tracheobronchial compression and feeding difficulties.^2^
